# Talpa: Or the Chronicles of a Clay Farm

**Published:** 1853-12

**Authors:** 


*Talpa: or the Chronicles of a Clay Farm. An Agricultural Fragment.* By C. W. H. With an Introduction and Notes, by LEWIS F. ALLEN. To which are added two Prize Essays on Tile Drainage. "*** ridentem dicere verum." Buffalo: Danforth, Hawley & Co.

Scattered throughout the diocese of our Journal, and no doubt furnishing to it a goodly share of its subscription list, are a class of farmer doctors, whose success in medicine has enabled them to add the independence of farm ownership, to the drudgery of practical medicine. To these men—these thought-free farmers—we commend the Chronicles of a Clay Farm.

The book is written in a Sartor Resartus sort of style, and the dry details of agriculture are enlivened by a keen, but seemingly unconscious wit, and adorned by the illustrations of a mind well stored with classical learning. Sundry little wood-cuts at the end of chapters are remarkably spirited and suggestive.

The Chronicles form a moderate single volume, neatly bound, and cleanly printed. He who purchases will find it a good investment in both a literary and scientific point of view. S. B. H.

For sale by DANFORTH, HAWLEY & Co.

